# The Effect of Health-Facility Admission and Skilled Birth Attendant Coverage on Maternal Survival in India: A Case-Control Analysis

**DOI:** 10.1371/journal.pone.0095696

**Published:** 2014-06-02

**Authors:** Ann L. Montgomery, Shaza Fadel, Rajesh Kumar, Sue Bondy, Rahim Moineddin, Prabhat Jha

**Affiliations:** 1 Centre for Global Health Research, Li Ka Shing Knowledge Institute, St. Michael Hospital, Toronto, Canada; 2 School of Public Health, Post Graduate Institute of Medical Education, Chandigarh, India; 3 Dalla Lana School of Public Health, University of Toronto, Canada; 4 Department of Family and Community Medicine, University of Toronto, Canada; 5 The Institute for Clinical Evaluative Sciences, Toronto, Canada; Iran University of Medical Sciences, Iran (Islamic Republic Of)

## Abstract

**Background:**

Research in areas of low skilled attendant coverage found that maternal mortality is paradoxically higher in women who seek obstetric care. We estimated the effect of health-facility admission on maternal survival, and how this effect varies with skilled attendant coverage across India.

**Methods/Findings:**

Using unmatched population-based case-control analysis of national datasets, we compared the effect of health-facility admission at any time (antenatal, intrapartum, postpartum) on maternal deaths (cases) to women reporting pregnancies (controls). Probability of maternal death decreased with increasing skilled attendant coverage, among both women who were and were not admitted to a health-facility, however, the risk of death among women who were admitted was higher (at 50% coverage, OR = 2.32, 95% confidence interval 1.85–2.92) than among those women who were not; while at higher levels of coverage, the effect of health-facility admission was attenuated. In a secondary analysis, the probability of maternal death decreased with increasing coverage among both women admitted for delivery or delivered at home but there was no effect of admission for delivery on mortality risk (50% coverage, OR = 1.0, 0.80–1.25), suggesting that poor quality of obstetric care may have attenuated the benefits of facility-based care. Subpopulation analysis of obstetric hemorrhage cases and report of ‘excessive bleeding’ in controls showed that the probability of maternal death decreased with increasing skilled attendant coverage; but the effect of health-facility admission was attenuated (at 50% coverage, OR = 1.47, 0.95–1.79), suggesting that some of the effect in the main model can be explained by women arriving at facility with complications underway. Finally, highest risk associated with health-facility admission was clustered in women with education 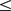
8 years.

**Conclusions:**

The effect of health-facility admission did vary by skilled attendant coverage, and this effect appears to be driven partially by reverse causality; however, inequitable access to and possibly poor quality of healthcare for primary and emergency services appears to play a role in maternal survival as well.

## Introduction

Maternal mortality in India has declined over the last twenty years, though it continues to represent 20% of the absolute number of 285 000 maternal deaths globally [1], [2]. Earlier studies have demonstrated an inverse relationship between the maternal mortality ratio (MMR) and skilled birth attendant coverage, either within-country over time or between-country comparisons [3], [4], but in a systematic review, Scott et al. found conflicting evidence whether attendance by a health professional reduced a woman’s risk of dying [5]. One interpretation is that, in areas of low skilled attendant coverage, maternal mortality is higher in women who do seek care because care may be more often sought for a critical obstetrical complication, and conversely, in areas of high coverage, women seek care in response to complications, as well as to avoid complications [6]–[11].

Using two Indian national surveys - the Sample Registration System and District Level Household Survey - we examined the effect of health-facility admission on maternal survival accounting for state level skilled attendant coverage as an effect modifier. Past studies have been limited to inter-country comparisons using a single national per cent coverage, or intra-country regional examination with single district level per cent coverage value. We hypothesized that as skilled attendant coverage increased, health-facility admission would begin to have a protective effect on maternal survival.

## Methods

### Study Design

We employed an unmatched population-based case-control study design to compare health-facility admission at any time in pregnancy, delivery or postpartum between cases of maternal death and control women who survived.

### Study Population

**Figure 1:**
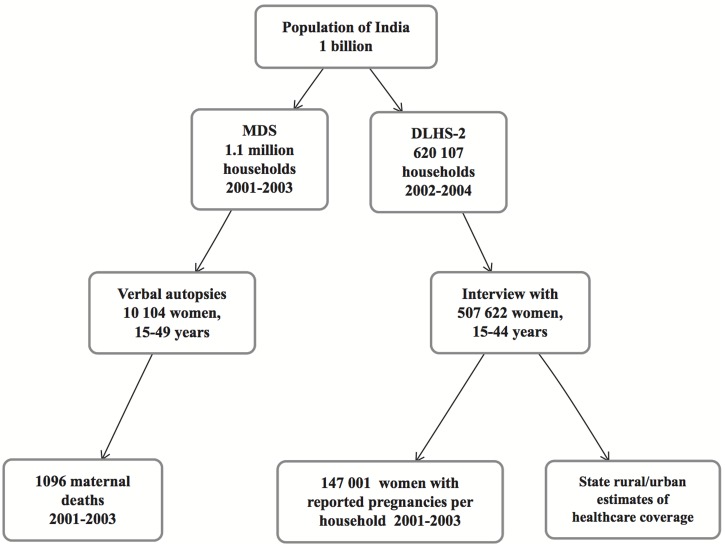
Flow diagram of sample population from national Indian datasets: MDS - Million Death Study, DLHS-2 - second round District Level Health Survey [18].

Cases were identified from India’s Million Death Study (MDS), a prospective, nationally representative population survey of mortality (Figure 1). Details of the methodology of the MDS are presented elsewhere [11]–[14]. In brief, the Registrar General of India monitors a nationally representative sample of 1.1 million households in the Sample Registration System (SRS). For every death occurring in these households from 2001–2003, a trained, non-medical survey or conducted a verbal autopsy with a relative or close acquaintance of the deceased. These records were assigned a cause of death using the International Classification of Diseases and Related Health Problems, 10th revision (ICD-10) [15] by two independent physicians [16]. For every deceased woman aged 15–49 years for whom the respondent answered affirmatively to the question *Was the deceased pregnant, 42 day post-abortion, or 42 days post-partum?* or whose record had been assigned an ICD-10 O-code (obstetric cause), the verbal autopsy report was reviewed by the study authors to confirm the woman’s death fulfilled the definition of a maternal death, and is further described elsewhere [17].

Controls were identified from the second round of India’s District Level Health Survey (DLHS-2). Details of the DLHS-2 are provided elsewhere and summarized here [18]. The DLHS-2 was conducted in 2002–2004 to monitor reproductive and child health coverage of services and their use. All married women aged 15–44 years in a nationally representative sample of households were interviewed on topics of reproductive and child health including antenatal care uptake, skilled birth attendance care, and healthcare utilization. Data on household water and sanitation, cooking fuel, housing type, and asset ownership were collected for each household. We selected as controls all women who reported any pregnancy outcome (live birth, stillbirth, spontaneous abortion, or induced abortion), limited to their most recent pregnancy, in the years 2001–2003.

We considered the whole sample of women as ‘at-risk’ of maternal death throughout the three-year survey period, drawn from the same base population. Each woman in our total study sample contributed information on their most recent pregnancy, and multiple births were recorded as one pregnancy.

### Study Data

#### Main exposure

The main exposure was binary (yes/no) for health-facility admission during the most recent pregnancy, for any reason. This refers to admission to any health-facility that has the capacity to manage primary care (delivery, induced abortion) or obstetric complications (Table 1). During the study period, India’s national healthcare strategy defined Community Health Centres (CHCs) and District Hospitals (DHs) as health-facilities with some basic emergency obstetric care as well as admission capacity. From the MDS, for all cases, informant report of admission to a health-facility was coded by one of the authors (ALM) from the open-ended narrative of the verbal autopsy using the validated Maternal Data Extraction Tool (M-DET) [19]. From the DLHS-2, for all controls, health-facility admission was ascertained through self-report of having sought obstetric care from a hospital (government or private) or a CHC [18].

**Table 1:** Definition of model variables.

Variable	Sample	Definition	Data
**Individual level Variable**			
Health-facility admission	Cases	Health-facility admission	MDS
	Controls	Health-facility admission to CHC or DH	DLHS
Antenatal care	Cases	Number of antenatal visits reported by respondent	MDS
	Controls	Number of antenatal visits reported by woman	DLHS
Antenatal care (Yes/No)	Cases	Report of one or more antenatal visits	MDS
	Controls	Report of one or more antenatal visits	DLHS
Education	Cases	Years of education received	MDS
	Controls	Years of education received	DLHS
Age	Cases	Age in years	MDS
	Controls	Age in years	DLHS
**District level variable** 1			
% households at low standard of living		Principal-component scoring of 12 variables including household water and sanitation,cooking fuel, housing type, and assetsownership	DLHS
**State level variable** 1			
% skilled attendant coverage		Reported births attended by doctor, ANM, nurse, LHV	DLHS

1 Applied to cases and controls; MDS - Million Death Study; DLHS - District Level Household Survey round 2; CHC - Community health centre; DH - District hospital; ANC - antenatal care; TT - tetanus toxoid vaccination; ANM - auxiliary nurse midwife; LHV - lady health visitor.

#### Covariates

**Figure 2:**
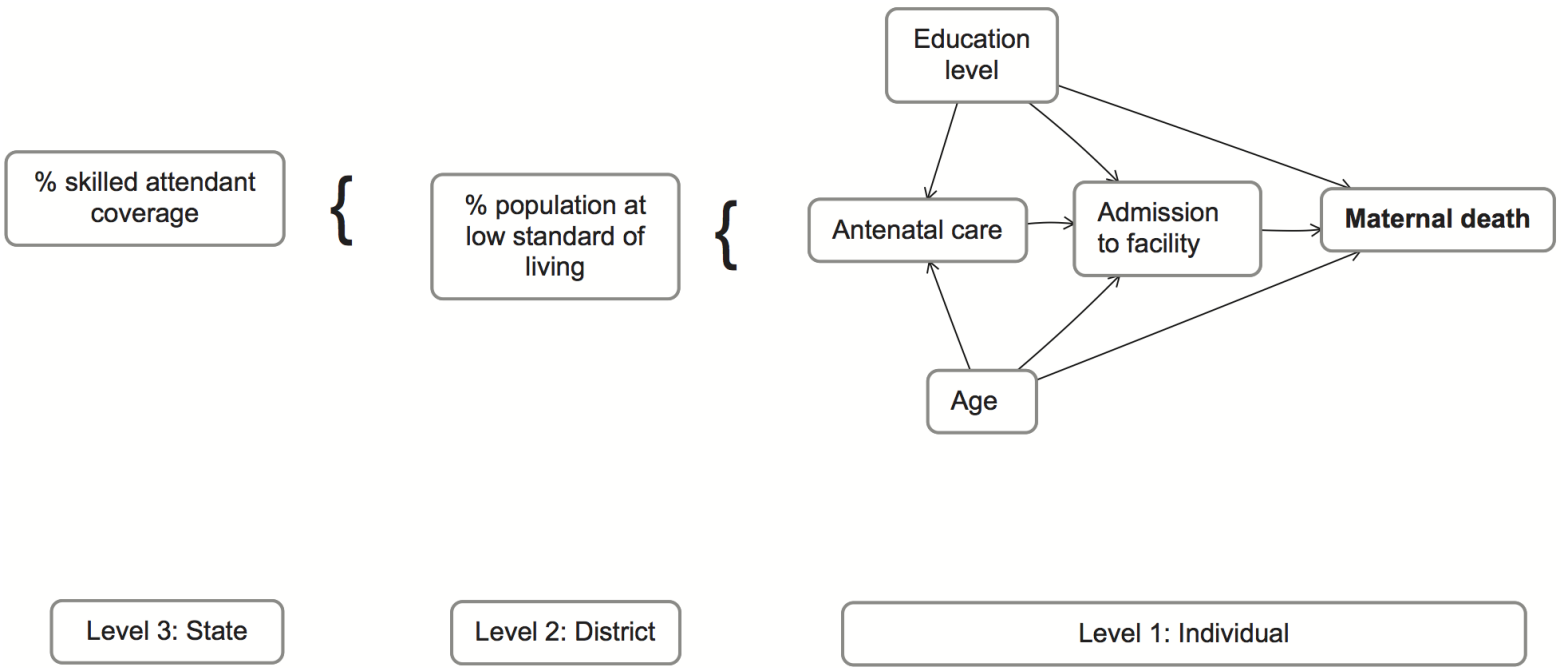
Conceptual framework of factors associated with maternal survival.

Covariates were selected a priori, having been identified in the literature to be associated with both maternal survival and obstetric access, and were selected based on the availability of relevant data in the surveys from which the cases and controls were respectively drawn (Figure 2).

#### Individual level

Individual-level covariates were woman’s age, education level and number of antenatal care visits. Education was categorized as none, primary school (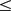
5 years), middle school (6–8 years), and secondary school (9–10 years) or above.

#### District level

We also included one district-level and one state-level covariate, matched to each woman’s place of residence using Pin (postal) codes for the MDS dataset (for cases) or district codes from the DLHS-2 (for controls). The proportion of households at a low standard-of-living in each district was collected from DLHS-2 reports, estimated from a principal-component scoring of 12 variables including household water and sanitation, cooking fuel, housing type, and assets ownership further described by the study authors [18].

#### State level

The proportion of births attended by skilled health personnel at home or in hospital in each state was collected from DLHS-2 reports for rural and urban areas separately. Skilled health personnel were defined as doctor, auxiliary nurse midwife, nurse or Lady Health Visitor [18].

To further describe our sample, we collected from the DLHS-2 the state-level proportions of female literacy and 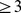 antenatal care visits, and the cesarean delivery rate; however, we did not include these variables in our models due to their collinearity with skilled birth attendant coverage. We note that skilled attendant coverage is a surrogate marker of a number of unmeasured population factors (female literacy, contraceptive prevalence, treatment resources, quality of the skilled healthcare provider, facility density, health care expenditure, transport access and decentralized services with efficient referral network [20]–[22]).

In order to consider fewer levels and to reduce the complexity, and computational intensity of the model, we excluded all but one woman from each household at random under the assumption that health-facility admission within households is highly correlated and additional women would add limited information.

### Statistical Analysis

We conducted bivariate analyses comparing health-facility admission between cases and controls over each level of the individual-, district- and state-level variables. We estimated the predicted probability of maternal death for women who were and were not admitted to a health-facility across the continuum of state-level skilled attendant coverage using a random effects logistic regression model in which each woman’s individual-level information is nested within districts within states. We report the adjusted OR for maternal death associated with health-facility admission at the mean national level of skilled attendant coverage, which was 50% at the time of the study period.

We constructed a random effects model based on the conceptual framework (Figure 2), incorporating an interaction term between health-facility admission and skilled attendant coverage (Appendix A in File S1). We checked for goodness-of-fit using Hosmer Lemeshow test statistic, the Loess curve, and scalar comparisons of BIC. We tested for the significance of the random intercept variance using the likelihood ratio test comparing cross-district and cross-state level variation. We estimated the intraclass correlation coefficient, *rho* (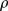), for maternal death for district- and state-level, with 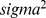 (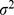) as the random-intercept variance [23].

We repeated the analysis of this main model using inverse probability weighting method to account for a woman’s propensity to seek care [24], [25] (Appendix C in File S1).

Of the total study sample, 6% of women were missing data for individual-level variables for either health-facility admission, antenatal care, age, or education level. We imputed missing values using multiple imputation by chained equations. We generated comparable estimates for imputed and complete case multivariate analysis (data not shown); therefore we assumed the missingness to be at random and would not bias the parameter estimates and all logistic regression results are presented for complete case analysis [26].

We further explored two alternative causal pathways not captured in our main model. For each, we estimated the predicted probabilities of death across the continuum of state-level skilled attendant coverage and the OR for mortality at a state-level coverage of 50%, using the same random effects model as our main model.

In the first analysis, we additionally considered quality of care to be a factor that may influence maternal survival using the WHO definition of quality of care [27]. We hypothesized that women attending a health-facility for delivery (so-called ‘booked women’) might have poorer outcomes compared to those women who stayed at home to deliver. Among cases, we included women whose verbal autopsy reported that admission to a health-facility was used as the ‘planned place of birth’ [19]. Among controls we included women who reported that a health-facility was used for delivery. In our second sub-analysis, we aimed to investigate the hypothesis that women with obstetric complications by creating a more comparable group who were more likely to be admitted to a health-facility than other women and were also more likely to die than other women, irrespective of whether they had been admitted to a health-facility. We therefore restricted this analysis to cases who died of obstetric hemorrhage (representing the largest single cause of death among all cases in the total study population (n = 296, 25% of maternal deaths)) and to controls who reported ‘excessive bleeding’ as a complication they experienced in either pregnancy, delivery, or up to 6 weeks postpartum (n = 17 093, 13% of controls). Report of excessive bleeding has been shown to be an indicator with relatively high sensitivity and specificity for an obstetric complication compared with other symptoms (e.g. puerperal sepsis, hypertensive disorders of pregnancy); and there is evidence in the literature that reports of vaginal bleeding would likely lead to care seeking in the Indian context [28]–[30]. Finally, in a third sub-analysis, we used linear combinations of coefficients of the main regression model to estimate the OR of death in women who used obstetric care in educated and/or urban women, relative to rural uneducated women who did not, accounting for the interaction of obstetric access with skilled attendant coverage, at state-level skilled attendant coverage of 50%.

All analyses were conducted using Stata/SE (StataCorp. 2011. Stata Statistical Software: Release 12. College Station, TX: StataCorp LP).

### Ethics

This study was approved by the Institutional Review Board of the Postgraduate Institute of Medical Education and Research (Chandigarh, India), the Indian Council of Medical Research, the Indian Health Ministry’s Screening Committee and the Institutional Review Board of St. Michael’s Hospital (Toronto, Canada).

## Results

Our total sample included 148 097 women (1096 cases of maternal deaths and 147 001 surviving control women) in 593 districts in 35 states. Given the low frequency of maternal death, the estimated odds ratio is approximately equal to the relative risk [31], [32].

Forty percent of the sample was admitted to a health-facility for either primary or emergency obstetric care (41% of cases and 38% of controls). These women tended to be younger, with a higher education, in receipt of antenatal care in the pregnancy, living in high income states, and from urban areas (Table 2). Women who had been admitted to a health-facility tended to be from districts that had a lower proportion of households at a low standard of living and from states that had higher skilled attendant coverage, female literacy, antenatal care uptake, and a higher cesarean delivery rate. The proportion of skilled attendant coverage ranged from 15–99%, with half the sample living in areas of coverage ranging from 24–60% (interquartile range (IQR)). The median coverage in the poorer and richer states was 24% (IQR 21–50%) and 59% (46–84%), respectively. Similarly, the median coverage in rural and urban areas was 30% (21–50%) and 69% (54–85%), respectively.

**Table 2:** Sample characteristics of women, 15–49 years (n = 148 097).

		Cases^a^		Controls^b^	
Individual level		n	%	n	%
Age (years)	15–19	112	10.2	10 363	7.1
	20–24	290	26.5	48 631	33.1
	25–29	210	19.2	48 211	32.8
	30–34	183	16.7	25 574	17.4
	35–39	118	10.8	10 728	7.3
	40–49	55	5.0	3 476	2.4
	Missing	128	11.7	18	0.0
Education	None	678	61.9	72 953	49.6
	Primary	181	16.5	17 497	11.9
	Middle	96	8.8	19 793	13.5
	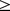 Secondary	69	6.3	36 758	25.0
	Missing	72	6.6	0	0.0
Religion	Hindu	790	72.1	111 044	75.5
	Muslim	157	14.2	18 919	12.9
	Other	71	6.5	15 408	10.5
	Missing	78	7.2	1 630	1.11
Number of antenatal visits	0	264	24.1	48 288	32.9
	1	85	7.8	10 800	7.4
	2	154	14.0	21 272	14.5
	3	120	11.0	17615	12.0
	4	68	6.2	9560	6.5
	5	40	3.6	8161	5.6
	6	51	4.7	7434	5.1
	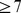	51	4.7	15 638	10.6
	Missing	262	23.9	8233	5.6
Health-facility admission^c^	Yes	453	41.3	56 627	38.52
	No	585	53.4	86 769	59.0
	Missing	58	5.3	3 605	2.5
Reported complications	Yes	1096	100.0	85 054	57.9
	No	0	0.0	61 947	42.1
Excessive bleeding	Yes	298	27.2	17 093	11.6
	No	798	72.8	129 908	88.4
State^d^	Low-income	723	70.0	82 764	56.3
	High-income	373	34.0	64 237	43.7
Place of residence	Rural	992	90.5	104 212	70.9
	Urban	104	9.5	42 789	29.1
**District level** (median, IQR)					
% household with low standardof living^e^		56.6	41.1–65.2	52.9	34.1–64.3
**State level, by rural urban**(median, IQR)					
% skilled attendant (SBA)coverage^f^		24.0	21.0–50.0	44.0	24.0–61.0
% full antenatal care coverage^g^		35.0	19.4–57.5	41.4	26.6–66.5
% cesarean delivery rate		2.6	2.3–6.0	5.3	2.3–9.3
% female literacy rate		42.8	40.4–57.3	55.3	40.4–70.0

Datasource: Indian MDS 2001–2003 and DLHS-2.

a Maternal deaths (cases).

b Surviving pregnant or postpartum women (controls).

c Health-facility admission (cases) Health-facility admission to community health centre or district hospital (controls).

d Low-income states Assam, Bihar, Chhattisgarh, Jharkhand, Madhya Pradesh, Orissa, Rajasthan, Uttar Pradesh, and Uttarakhand.

e DLHS-2 Standard of living index [29].

f Proportion of deliveries with a skilled birth attendant, at home or in a health-facility.

g Proportion of women who receive 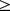
3 antenatal visits in their pregnancy.

**Figure 3:**
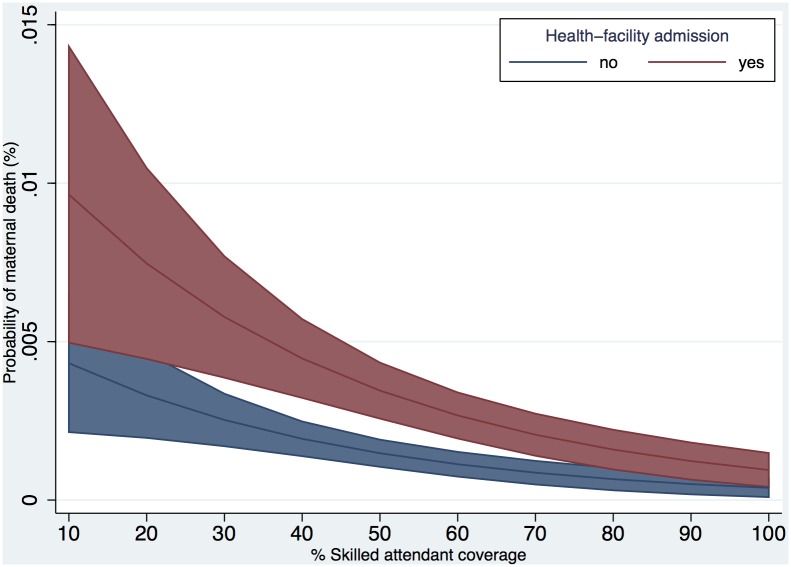
Predicted probability of maternal death by % skilled attendant coverage over health-facility admission. Datasouce: Indian MDS 2001–2003 and DLHS-2. Regression models adjusted for: fixed effects - receipt of antenatal care, age, 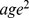, education, place of residence (rural/urban), district level standard of living, and interaction between health-facility admission and skilled attendant coverage; random effects - district cluster, state cluster. Presented with 95% CI. Women (n = 139 321) in districts (n = 593) in states (n = 35); random effects 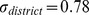, 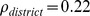, 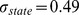, 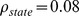.

In the main model, the probability of maternal death decreased with increasing skilled attendant coverage, among both women who were and were not admitted to a health-facility (Figure 3), however, within the interquartile range (24–60%) of coverage, the risk of death among women who were admitted to a health-facility was higher than among those women who were not; while with higher levels of coverage, there was no effect of health-facility admission. We also compared point estimates of the main model with the inverse probability weighting method, to account for a woman’s propensity to access obstetric care accounting for known covariates, and estimated a comparable OR of 2.78 (95% CI 2.21–3.51) at 50% skilled attendant coverage (Appendix C in File S1). Among those who were admitted to a health-facility, there was an eight-fold decrease in the probability of maternal death as skilled attendant coverage increased from 10 to 80%, and a three-fold decrease for those who were not admitted to a health-facility. There was substantial variation in maternal deaths across districts and states (provided in Figure 3). There was a moderate level of district clustering (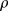), which suggests that 20% of the total variation is related to the heterogeneity across districts. Coefficients and p-values of the model are provided in Appendix B in File S1, Table S1.

**Figure 4:**
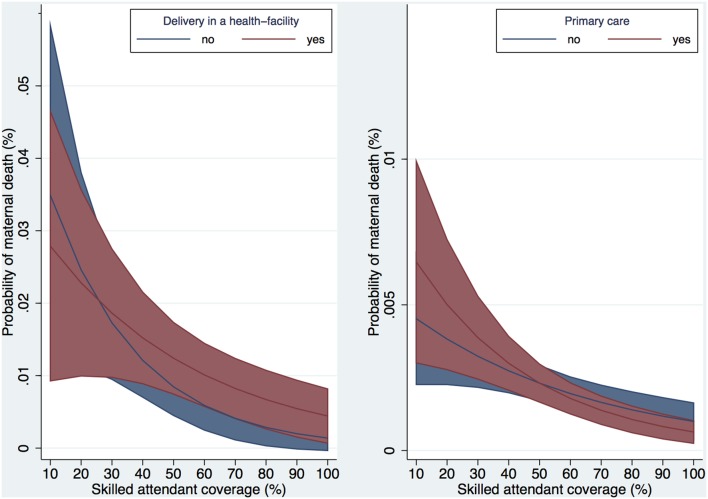
Predicted probability of maternal death by % skilled attendant coverage over (4a) delivery in health-facility and (4b) health-facility admission in sub-population of obstetric hemorrhage. Datasouce: Indian MDS 2001–2003 and DLHS-2. Adjusted for receipt of antenatal care, age, 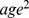, education, place of residence (rural/urban), district level standard of living, and interaction between skilled attendant coverage and (4a) delivery in a health-facility or (4b) health-facility admission. Presented with 95% CI. (4a) Women (n = 139 417) in districts (n = 593) in states (n = 35); random effects 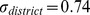 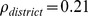 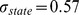 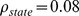 (4b) Women (n = 17 391) in districts (n = 593) in states (n = 35); random effects 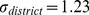, 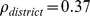, 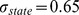, 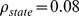.

**Figure 5:**
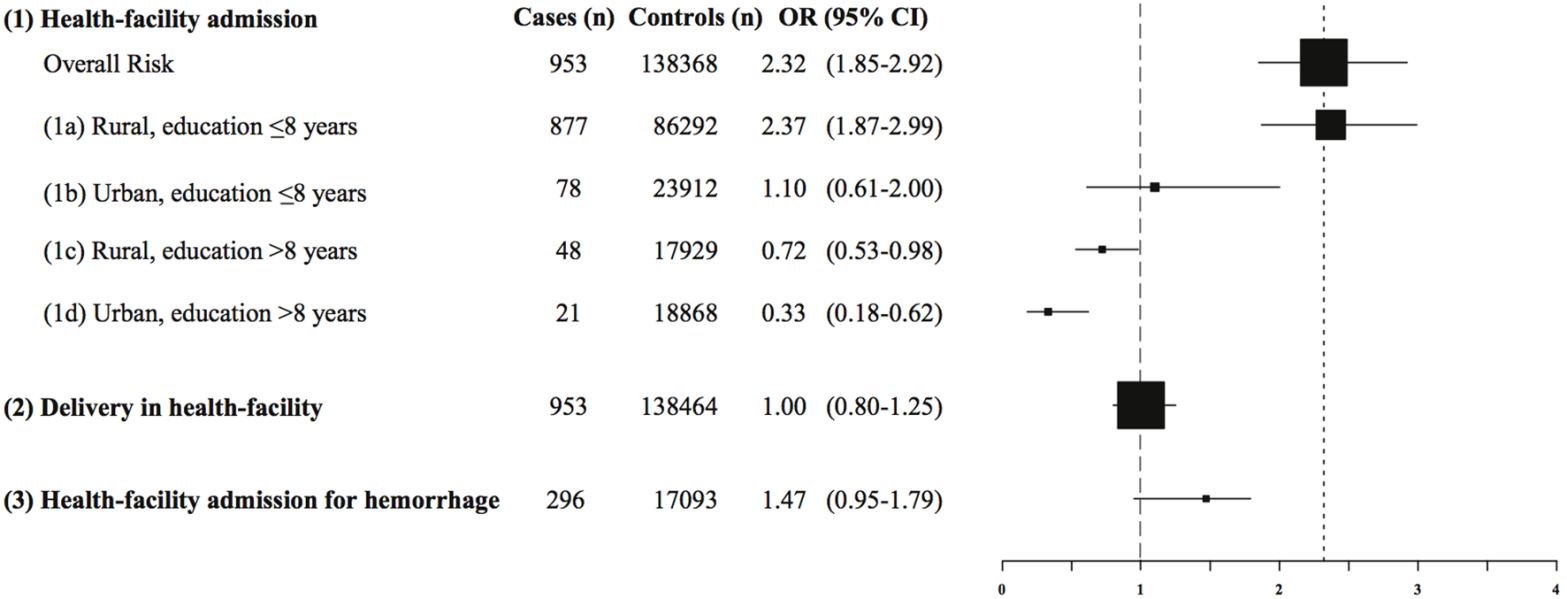
Odds ratio of maternal death by exposure at 50% skilled attendant coverage. Datasource: MDS and DLHS-2 2001–2003. All regression models adjusted for: fixed effects - receipt of antenatal care, age, 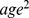, education, place of residence (rural/urban), district level standard of living, and interaction between main exposure and skilled attendant coverage; random effects - district cluster, state cluster. (1) Main exposure: health-facility admission (1a–1d - linear combinations of coefficients after estimation of this regression model) (2) Main exposure: delivery in health-facility (Routine) (3) Main exposure: health-facility admission; main outcome: hemorrhage in subpopulation.

In the secondary analysis, the probability of maternal death decreased with increasing skilled attendant coverage among both women who delivered in a health-facility and those who delivered at home (Figure 4) but there was no association of delivery in a health-facility with mortality risk. The graph illustrates a cross-over such that below 50% coverage, delivery in a health-facility is a risk of mortality; whereas above 50% coverage, delivery in a health-facility is protective. Statistical interaction between skilled attendant coverage and primary care was considered and the appropriate interaction terms were added to the regression models, and tested for both interaction on the additive and multiplicative level, and neither test was statistically significant. Similarly, in our subpopulation analysis of obstetric hemorrhage cases and report of ‘excessive bleeding’ in controls, we again found that the predicted probability of maternal death decreased with increasing skilled attendant coverage (Figure 4); but the effect of health-facility admission was attenuated compared to our main analysis of the full study population. The graph illustrates a cross-over such that below 24% coverage, health-facility admission is protective for mortality; whereas above 24% coverage, health-facility admission is a risk. Statistical interaction between skilled attendant coverage and health-facility admission was considered and the appropriate interaction terms were added to the regression models, and tested for both interaction on the additive and multiplicative level, and neither test was statistically significant. Finally, health-facility admission was a risk for rural women with 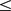
8 years of education (Figure 5). The OR for each of these models is summarized in Figure 5 at 50% skilled attendant coverage.

## Discussion

Our study of the effect of women’s healthcare use during pregnancy and delivery on the risk of maternal mortality in India demonstrated that effect of health-facility admission varies by population and regional skilled attendant coverage.

Controlling for other factors, in areas with 50% skilled attendant coverage, representing the average coverage in India at the time of the survey (2001–2003), the probability of maternal death was significantly higher in women who were admitted to a health-facility than in women who were not. In areas with higher coverage, the predicted probability of maternal death was lower overall, and health-facility admission showed no association with maternal mortality risk.

If poor quality of care was driving this effect of higher mortality risk among women who were admitted to a health-facility, then we would also expect that women who delivered in a health-facility would have poorer outcomes than women delivering at home. Instead, there was no statistical association between delivery in a health-facility and risk of maternal death, indicating that poor quality of care does not wholly explain our results. Further, we describe a cross-over effect at 50% skilled attendant coverage, where delivery in a health facility begins to show a protective effect, albeit not statistically significant. Secondly, we accounted for a prognostic factor in a subpopulation who reported vaginal bleeding, in order to account for confounding of disease severity causing both care-seeking and death (referred to as ‘home birth bias’ [33], self-selection bias [6], [34], reverse causality [35], or common cause confounding [36]). We demonstrated an attenuation of the measure of effect, suggesting that some of the effect in the main model can be explained by women arriving at the facility with complications underway. Thirdly, we did find that health-facility admission was associated with higher relative risk of maternal mortality in women with lower compared to higher education, at average skilled attendant coverage (50%).

We took several steps to address the reverse causality thought to be at play. We accounted for skilled attendant coverage as well as biases operating through (delivery in a health-facility and hemorrhage events) and the individual woman’s characteristics (SES), using random effects logistic regression modelling and a novel application of inverse probability weighting (Appendix C in File S1, Figures S1–S3). We hypothesized that accounting for skilled attendant coverage would address the limitations of past studies, which found a higher odds of death in those women who accessed obstetric care in areas of low coverage [6], [7], [37], [38]. We hypothesized that as skilled attendant coverage increased, health-facility admission would begin to have a protective effect on maternal survival, yet we did not find a protective effect of health-facility admission in areas of high coverage and we propose that the reasons for this could be methodological, or a reflection of inequitable access and poor quality of care, or a mix of both of these.

The study limitations are in the form of differential misclassification of health-facility admission, which may have occurred since proxy respondents were interviewed for cases, while control women were interviewed directly. We conducted a sensitivity analysis, varying the estimated sensitivity and specificity of reported obstetric access for cases and controls and we replicated the same crude point estimate, providing some reassurance that potential differential misclassification of exposure was not a major issue (Appendix D in File S1, Figure S4, for further discussion). In addition to misclassification, health-facility admission is a crude proxy for what is really undefined multiple versions of treatment as quality and availability of care likely varied by geographical and SES distribution [39]. This error in exposure measurement may have biased the results towards null in areas of high coverage. Finally, maternal mortality is rare, and in order to show a difference in the OR in the highest skilled attendant coverage quartile, a large sample size (tens of thousands of cases) would be required.

Second, we considered inequitable access and poor quality of care as an explanation for our results. India has one of the highest out-of-pocket expenditure on health in the world and 42% of the population in poverty [40]–[43]. Of 54 countries reviewed for distribution of antenatal care and skilled birth attendance by wealth index, India was categorized in the top 10 of ‘highly inequitable’ countries [44]. Several Indian studies have shown that women of low SES use fewer services, and the services they do use is of poorer quality [45]–[51]; conversely, Tamil Nadu state has improved maternal outcomes and has credited programs specific to increasing access to care for marginalized women [52], [53]. In the analysis using the alternative hypothesis using delivery in a health-facility as the exposure, there was no statistically significant effect on maternal death, suggesting that quality of care does not wholly explain the effect, as women who delivered in a health-facility did not have better outcomes in areas of low coverage, and no statistically significant protective effect in areas of high coverage. As well, in the subpopulation analysis of hemorrhage events, there was an attenuation of effect, suggesting that some of the effect in the main analysis is due to women first accessing services in a critical condition. And finally, for women with 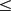
8 years of education, health-facility admission was an excess risk for this subpopulation of the sample. These points suggests that there is a clustering of both poor outcomes and poor care among the poorest women, even in areas of high coverage [54].

Our study provides a baseline for further research on the effect of facility-based obstetric care on maternal survival. In 2005, the Indian government introduced a conditional cash-transfer policy to increase skilled birth attendant uptake, which has resulted in some success in increasing coverage [55], [56], though its impact on maternal and neonatal mortality appears to have been limited [54], [57]–[60]. Further, the National Rural Health Mission policy in 2005 has led to a significant increase in the number and emergency obstetric care (EmOC) capacity of CHCs and DHs in rural areas of poorer states.

Our results suggest that increasing skilled attendant coverage is a necessary, if not sufficient, condition for decreasing maternal mortality. However, we caution that coverage is a surrogate marker of a number of unmeasured population factors also requiring government investment [20]–[22]. Health services, such as obstetric care, can contribute to better outcomes for women and their newborns, and have shown to contribute to a reduction in inequity in health, especially when primary care services are explicitly considered [21], [53], [61]–[63]. The effect of health-facility admission did vary by skilled attendant coverage, and this effect appears to be driven partially by reverse causality; however, inequitable access to and possibly poor quality of healthcare for primary and emergency services appears to play a role in maternal survival as well.

## Supporting Information

Figure S1 **Standardized difference of means before and after weighting.** Datasouce: Indian MDS 2001–2003 and DLHS-2. Pnc_edu_v3 - interaction of receipt of antenatal care (ANC) and education; d_fullanc_sba - interaction of district level % population in receipt of 3 ANC visits and % of skilled birth attendance; d_low_sli V02 - district level of % of households living at low standard of living; rural - place of residence (rural urban); eaga - low income states (yes/no); age - age (years); rel_tri - religion (Hindu, Muslim, other).
(TIFF)

Figure S2 **Predicted probability of death by % skilled attendant coverage over health-facility admission, using inverse probability weighting method.** Datasouce: Indian MDS 2001–2003 and DLHS-2. Inverse probability weighting accounts for interaction between health-facility admission and skilled attendant coverage. Presented with 95% CI, assuming independence.
(TIFF)

Figure S3 **Odds ratio of death of random effects model and inverse probability weighted model at 50% skilled attendant coverage.** Datasource: MDS and DLHS-2 2001–2003. Model adjusted for: fixed effects - receipt of antenatal care, age, *age* ^2^, education, place of residence (rural/urban), district level standard of living, and interaction between health-facility admission and skilled attendant coverage; random effects - district cluster, state cluster. Inverse probability weighting accounts for interaction between health-facility admission and skilled attendant coverage. Presented with 95% CI, assuming independence.
(TIFF)

Figure S4 **Assessment of misclassification bias.** Datasource: MDS and DLHS-2 2001–2003. Crude odds ratio of maternal death given health-facility admission. Estimation of stochastic differential error in which health-facility admission classification for cases is 80–88% sensitivity and 90–95% specificity, and health-facility admission classification for controls is 90–95% sensitivity and 90–95% specificity; 2000 simulations.
(TIFF)

File S1 (PDF)
