# Supplementary figures and images for: The Effect of Health-Facility Admission and Skilled Birth Attendant Coverage on Maternal Survival in India: A Case-Control Analysis
Source: PLoS One. 2014 Jun 2;9(6):e95696. doi: 10.1371/journal.pone.0095696 (PMC4041636; doi:10.1371/journal.pone.0095696)

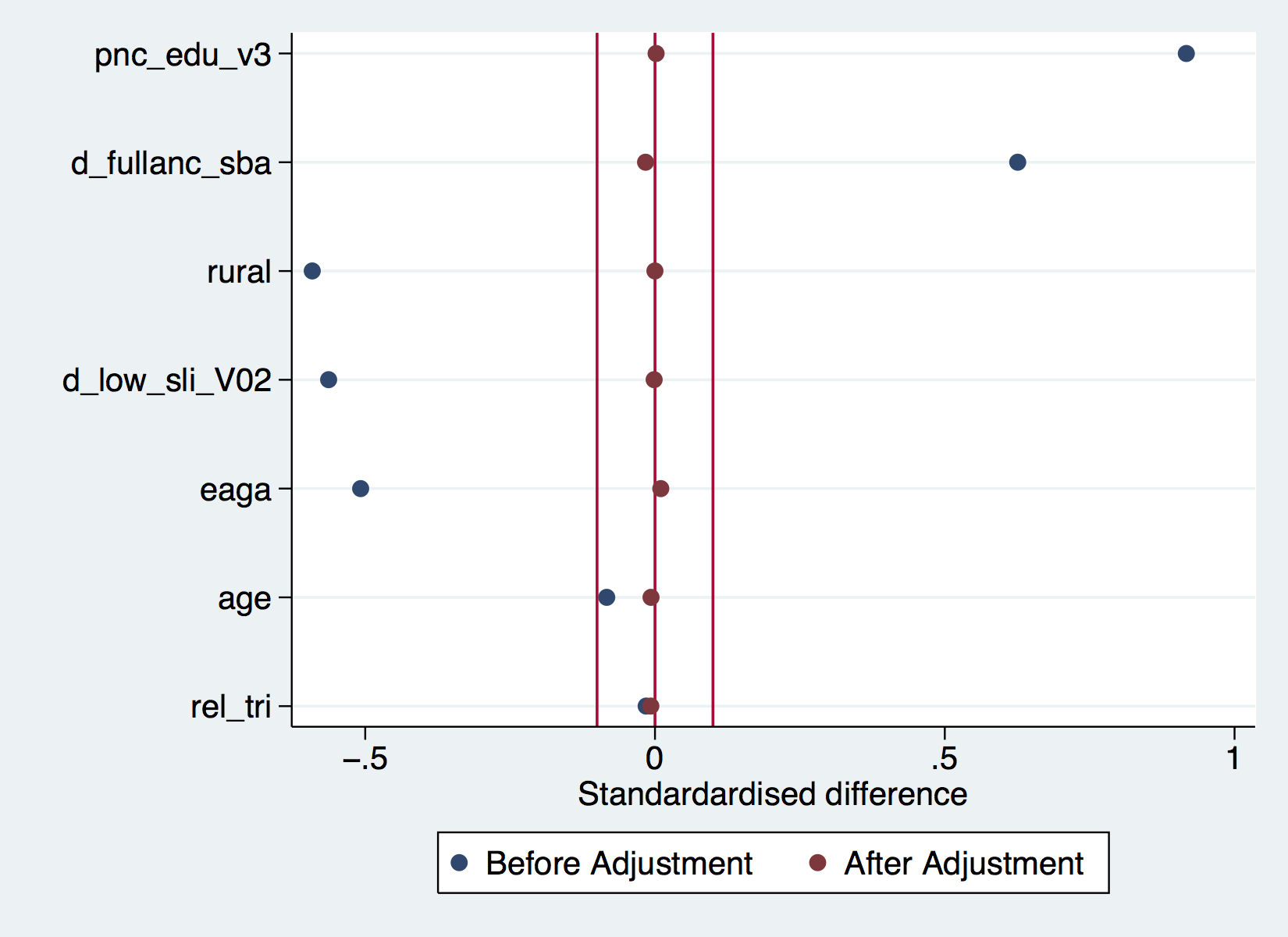

Supplement: Figure S1 — Standardized difference of means before and after weighting. Datasouce: Indian MDS 2001–2003 and DLHS-2. Pnc_edu_v3 - interaction of receipt of antenatal care (ANC) and education; d_fullanc_sba - interaction of district level % population in receipt of 3 ANC visits and % of skilled birth attendance; d_low_sli V02 - district level of % of households living at low standard of living; rural - place of residence (rural urban); eaga - low income states (yes/no); age - age (years); rel_tri - religion (Hindu, Muslim, other). (TIFF) [file pone.0095696.s001.tiff]

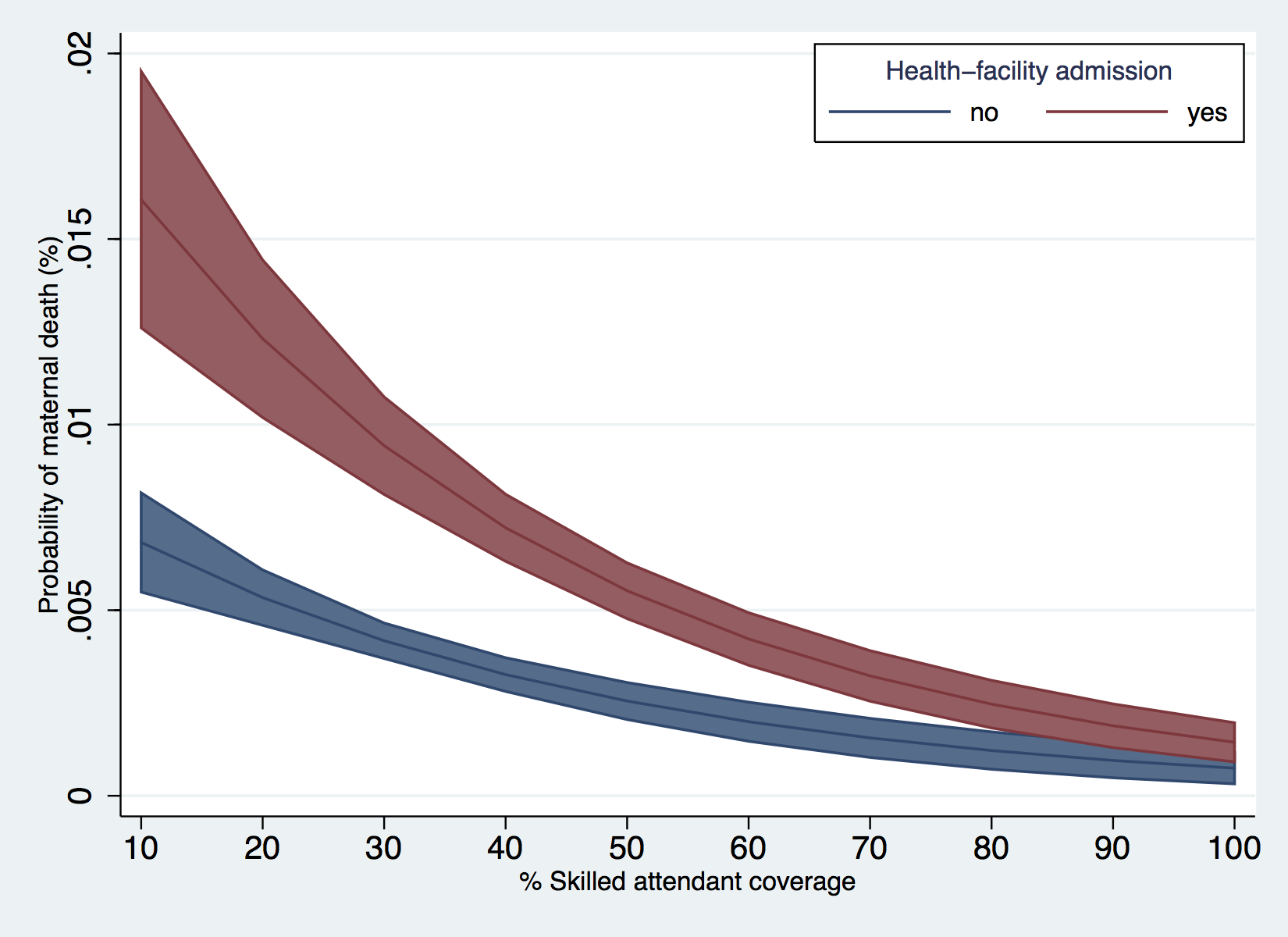

Supplement: Figure S2 — Predicted probability of death by % skilled attendant coverage over health-facility admission, using inverse probability weighting method. Datasouce: Indian MDS 2001–2003 and DLHS-2. Inverse probability weighting accounts for interaction between health-facility admission and skilled attendant coverage. Presented with 95% CI, assuming independence. (TIFF) [file pone.0095696.s002.tiff]

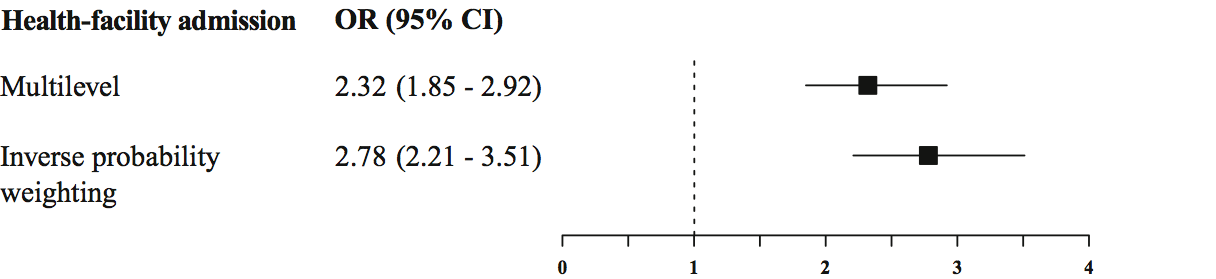

Supplement: Figure S3 — Odds ratio of death of random effects model and inverse probability weighted model at 50% skilled attendant coverage. Datasource: MDS and DLHS-2 2001–2003. Model adjusted for: fixed effects - receipt of antenatal care, age, age 2, education, place of residence (rural/urban), district level standard of living, and interaction between health-facility admission and skilled attendant coverage; random effects - district cluster, state cluster. Inverse probability weighting accounts for interaction between health-facility admission and skilled attendant coverage. Presented with 95% CI, assuming independence. (TIFF) [file pone.0095696.s003.tiff]

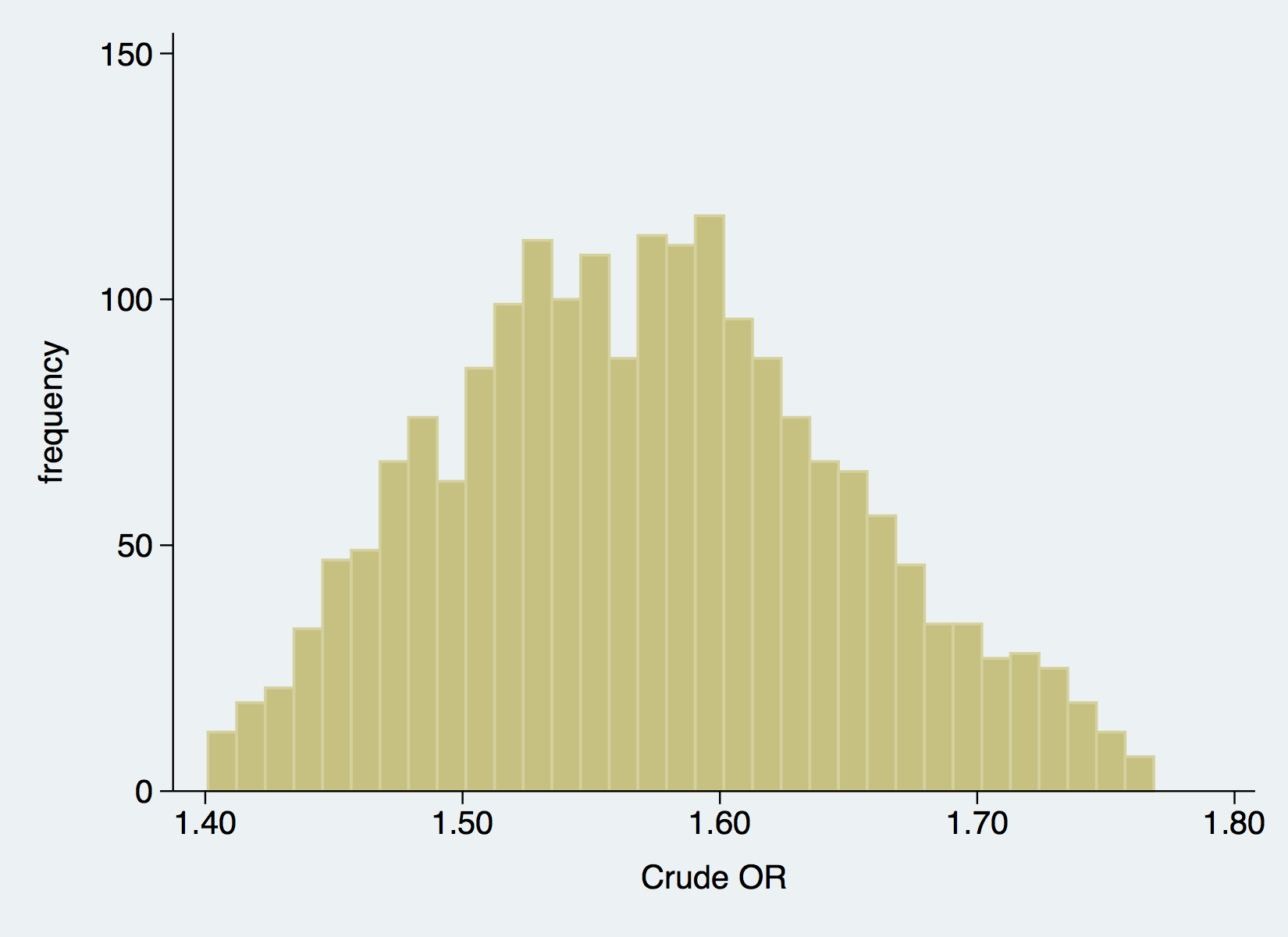

Supplement: Figure S4 — Assessment of misclassification bias. Datasource: MDS and DLHS-2 2001–2003. Crude odds ratio of maternal death given health-facility admission. Estimation of stochastic differential error in which health-facility admission classification for cases is 80–88% sensitivity and 90–95% specificity, and health-facility admission classification for controls is 90–95% sensitivity and 90–95% specificity; 2000 simulations. (TIFF) [file pone.0095696.s004.tiff]
