# Supplementary material for: The Effect of Health-Facility Admission and Skilled Birth Attendant Coverage on Maternal Survival in India: A Case-Control Analysis
Source: PLoS One. 2014 Jun 2;9(6):e95696. doi: 10.1371/journal.pone.0095696 (PMC4041636; doi:10.1371/journal.pone.0095696)
Supplement: File S1 — (PDF) [file pone.0095696.s005.pdf]

Appendix: The effect of health-facility admission on maternal survival

Ann L. Montgomery[1*],
1 Centre for Global Health Research, Li Ka Shing Knowledge Institute, St. Michael Hospital, Toronto, Canada
∗ E-mail: cghr@smh.on.ca

Supplementary Appendix A - Random effects model

In our notation, we indicate i for the ith individual in the jth district in the kth state. We further assume that the logit of the conditional probability of maternal death (π_{ijk}) in the ith women in the jth district in the kth state given the random effects satisfies this main model:

$$logitPr(y_{ijk} = 1|x_{ijk}, \zeta_{jk}, \zeta_k) = \beta_1 + \beta_2 x_{2ijk} + \cdots + \beta_7 x_{7,k} + \beta_8 x_{2ijk} x_{7,k} + \zeta_{jk} + \zeta_k \quad (1)$$

Where the $\beta_8 x_{2ijk} x_{7,k}$ represents the interaction term between health-facility admission and skilled attendant coverage, the segment $\beta_1 + \beta_2 x_{2ijk} + \cdots + \beta_7 x_{7,k} + \beta_8 x_{2ijk} x_{7,k}$ represents the fixed effects, and the segment $\zeta_{jk} + \zeta_k$ represents the random effects. We estimated the total variance in maternal death that can be attributed to between-district (Equation 2) and between-state heterogeneity (Equation 3) using the conditional intraclass correlation coefficient, ρ [1].

$$\rho_{(district,state)} = \frac{\sigma_d^2 + \sigma_s^2}{\sigma_d^2 + \sigma_s^2 + \frac{\pi^2}{3}} \quad (2)$$

$$\rho_{(state)} = \frac{\sigma_s^2}{\sigma_d^2 + \sigma_s^2 + \frac{\pi^2}{3}} \quad (3)$$

Where σ^2 is the random-intercept variance in a fully unconditional model, and where $\rho_{(district,state)} > \rho_{(state)}$ as women of same districts are more similar than women of the same state but different districts.

Supplementary Appendix B - Coefficients and p-values of main model

Table S1: Coefficients and p-values of main model (n=139 319)

Variable	Coefficient	p-value	95% CI
Health-facility admission	0.8213	0.0001	0.4275-1.2151
Receipt of antenatal care (Yes/No)	1.0423	0.0001	0.8335-1.2510
Age	-0.2593	0.0001	-0.3573- -0.1613
Age^2	0.0049	0.0001	0.0033-0.0066
Place of residence	-0.7988	0.007	-1.3795- -0.2181
Education	-1.1927	0.0001	-1.4290- -0.9565
% of population at low standard of living in district	-0.0004	0.917	-0.0081-0.0072
% skilled attendant coverage in state	-0.0159	0.031	-0.0305- -0.0014
Health-facility admission x coverage	0.0007	0.885	-0.0093-0.0108
Constant	-2.6667	0.001	-4.2884- -1.0451

Datasource: Indian MDS 2001-2003 and DLHS-2. Women (n=139 321) in districts (n=593) in states (n=35); random effects $\sigma_{district} = 0.78$, $\rho_{district} = 0.22$, $\sigma_{state} = 0.49$, $\rho_{state} = 0.08$.

Supplementary Appendix C - Alternative method: inverse probability weighting

Methods

Inverse probability (IP) weighting is alternative method to estimate treatment effects and to reduce the effect of self-selection bias. IP weighting accounts for a woman's propensity to seek care given known covariates [2, 3]. We used iterative forward stepwise regression to estimate a woman's probability of health-facility admission (Pr(health-facility admission)) given the individual covariates - age, education, receipt of antenatal care, religion, and rural residence; and the district level covariates (%) - households with low standard of living, skilled attendant coverage, and full antenatal care (≥3 antenatal visits). We constructed several of these models to find the optimal balance in the weighted sample between the exposed and unexposed groups (standardize difference in means ≤ 0.01). Using the optimal model, we then calculated each individual woman's IP weight. The IP weight is the inverse of the probability of receiving the treatment that the woman actually received (Equation 4 and Equation 5). We then conducted a weighted logistic regression model to estimate the effect of the main exposure (health-facility admission) on maternal survival, accounting for the state level proportion of skilled attendant coverage. We used Taylor series linearization to calculate sample standard errors however, a limitation of the IP weighting method is that it is unable to account for the clustering effect hierarchical data; thus, 95% confidence intervals for these estimates are biased and should be ignored.

All analyses were conducted using Stata/SE (StataCorp. 2011. Stata Statistical Software: Release 12. College Station, TX: StataCorp LP).

$$IPweight_{HF} = \frac{1}{Pr(HF)} \tag{4}$$

$$IPweight_{NoHF} = \frac{1}{1 - Pr(HF)} \tag{5}$$

where HF is health-facility admission

Results

We estimated the probability of treatment and achieved acceptable balance in the distribution of known covariates in treated and untreated individuals (Figure S1).

In the IP weighted sample, health-facility admission was a risk of maternal death at low levels of coverage, and did not confer a protective effect at high levels of coverage (Figure S2). Estimated odds ratio (OR) at 50% skilled attendant coverage is summarized in Figure S3 and compared to the random effects model.

Figure S1: Standardized difference of means before and after weighting

Datasouce: Indian MDS 2001-2003 and DLHS-2. pnc_edu_v3 - interaction of receipt of antenatal care (ANC) and education; d_fullanc_sba - interaction of district level % population in receipt of 3 ANC visits and % of skilled birth attendance; d_low_sli_V02 - district level of % of households living at low standard of living; rural - place of residence (rural urban); eaga - low income states (yes/no); age - age (years); rel_tri - religion (hindu, muslim, other).

Figure S2: Predicted probability of death by % skilled attendant coverage over health-facility admission, using inverse probability weighting method

Datasouce: Indian MDS 2001-2003 and DLHS-2. Inverse probability weighting accounts for interaction between health-facility admission and skilled attendant coverage. Presented with 95% CI, assuming independence.

Figure S3: Odds ratio of death of random effects model and inverse probability weighted model at 50% skilled attendant coverage

Datasource: MDS and DLHS-2 2001-2003. Model adjusted for: fixed effects - receipt of antenatal care, age, age^2, education, place of residence (rural/urban), district level standard of living, and interaction between health-facility admission and skilled attendant coverage; random effects - district cluster, state cluster. Inverse probability weighting accounts for interaction between health-facility admission and skilled attendant coverage. Presented with 95% CI, assuming independence.

Discussion

Using IP weighting method, we estimated that health-facility admission does not confer a protective effect on maternal survival in this context, achieving similar results as the random effects model in the main analysis.

The objective of IP weighting is to reduce the self-selection bias given the known covariates. The extent that the bias is reduced depends on the 'richness' or quality of the covariates used to estimate the IP weight. Like most population datasets, our datasets lack detailed descriptions of individuals (i.e. social, medical) which would better inform the IP weighting method. Nonetheless, in comparison to the random effects model, using the same data and a different method, we replicated the OR of maternal death conditional on the known covariates.

Supplementary Appendix D - Quantitative bias analysis: exposure misclassification

Methods

We estimated the impact of differential misclassification bias of health-facility admission on the OR estimate. Differential misclassification of health-facility admission may have occurred since proxy respondents were interviewed for cases, while control women were interviewed directly. This bias could stem from the different wording of the question in two different surveys, or due to very different experiences of the respondents introducing recall and social desirability bias. To explore this, we conducted a sensitivity analysis, differentially varying the estimated sensitivity and specificity of health-facility admission classification for cases and control [4, 5]. For cases, we estimated a sensitivity and specificity of 80-88% and 90-95% respectively, and for controls, a sensitivity and specificity of 90-95% and 90-95% respectively. We conducted a stochastic analysis from a uniform distribution for 2000 simulations. All analyses were conducted using Stata/SE (StataCorp. 2011. Stata Statistical Software: Release 12. College Station, TX: StataCorp LP).

Results

The median value for the simulated crude OR was 1.55 (95% of the crude values fell in the range of 1.40-1.80. (Figure S4), compared with a crude OR of 1.19 (95%CI 1.05-1.34) assuming no error in classification of health-facility admission, indicating that the direction of the effect may be further from the null estimate the less sensitive the health-facility admission classification was for the cases.

Figure S4: Assessment of misclassification bias

Datasource: MDS and DLHS-2 2001-2003. Crude odds ratio of maternal death given health-facility admission. Estimation of stochastic differential error in which health-facility admission classification for cases is 80-88% sensitivity and 90-95% specificity, and health-facility admission classification for controls is 90-95% sensitivity and 90-95% specificity; 2000 simulations.

References

1. Rabe-Hesketh S, Skrondal A (2012) Multilevel and longitudinal modeling using Stata: categorical responses, counts, and survival. College Station: Stata Press Publication, 3rd edition.

2. Austin PC (2011) A tutorial and case study in propensity score analysis: an application to estimating the effect of in-hospital smoking cessation counseling on mortality. Multivar Behav Res 46: 119-151.

3. Hernán MA, Robins JM (2013) Casual inference. New York: Chapman and Hall / CRC.

4. Lash TL, Fox MP, Fink AK (2009) Applying quantitative bias analysis to epidemiologic data: statistics for biology and health. New York: Springer, illustrated edition.

5. Dohoo IR, Martin SW, Stryhn H (2012) Methods in Epidemiologic Research. Charottetown: VER Incorporated.
